# Indents

**Published:** 1922-04

**Authors:** 


### INDENTS

**☞Brief Articles of Technical or Scientific Value will receive notice and comment. Contributions invited.**

THE ACCEPTED FILLING MATERIALS. In relative value the cast restoration will take its place as one of the two best at our command, these two being the gold foil filling and the cast inlay. The former, when properly made and when placed where it is indicated, has proved to be the most satisfactory and the most long-lived restoration. However, the inlay has the greatest value to the profession because it can be much more universally used. In the consideration of restorations, it might be well to digress a moment to consider the fundamental requisites for a filling material. (1) It should be an adaptable material by the use of which the restoration will restore the lost parts to complete functional contour, that is, reproduce the anatomy to the finest detail. (2) It must hermetically seal the cavity. (3) It must be of a constant form and free from shrinkage and expansion. (4) It must not deteriorate, corrode, or disintegrate in the mouth. (5) It must be hard and resistant to withstand occlusal and interproximal wear. (6) It should be aesthetic in appearance. (7) It must be of such a nature that it can be successfully placed in locations difficult of access. (8) It must be free from porosity, that is, non-absorbent, for an absorbent material will become saturated with bacterial products and putrefactive matter, thus causing infection of the dentin and pulp. (9) It should, to a certain extent at least, be a non-conductor of heat. (10) It must by a combination of these properties be capable of a long and satisfactory service. Gold foil we know will meet these requirements. The inlay probably will too, if properly made, but its case has not as yet been so well proved. The plastics, with the exception of amalgam, most emphatically will not. Even with amalgam long service is an exception and not the rule.

One of the plastics which absolutely fails to meet any of these requirements but that of aesthetics and ease of insertion has come into extensive use. This material is the silicate cement or so-called synthetic porcelain. Its use is not to be considered in really high-class practice.

Unless it is greatly improved it should be eliminated. These statements can be amply verified by any operator who will carefully study conditions and observe the serious effects and amount of pathology which its use entails. From the foregoing statements, we are amply justified in assuming that of the several methods and materials at hand the gold foil filling and the cast inlay restoration are by far the best. Each has its place and neither should be used to the exclusion of the other. As a general rule, the inlay is best suited for large, complicated restorations in the posterior teeth, and foil for the smaller restorations in the posteriors and for the great majority of anterior restorations.—G. M. Hollenback, in *Journal N. D. A.*

---

Multiple Extractions and Their Complications. Several clinical cases have come under the writer's observation within a period of a few weeks illustrative of the possible complications of multiple dental extractions, a problem in dental pathology which has been previously discussed in the editorial department of this publication. One of these cases was that of a young person suffering from a chronic malady in whom the progress of the disease appeared to have become arrested and the symptoms, objective and subjective, augured for as full a recovery as could be expected in such cases. Her dental adviser, following a radiographic examination, discovered eleven teeth the seat of unrecoverable apical involvements and recommended their extraction. With such a recommendation, in view of the extensiveness of the peridental and alveolar lesions, no fault is to be found; it was the only permissible solution of a complicated situation. The extractions were performed, all at one sitting, under conductive anesthesia, and in addition an extensive curettement of all diseased areas was carried out. A short time thereafter,—in fact only a few hours,—the patient suffered from a severe physical and mental collapse, for weeks thereafter her life seemed to hang by the slimmest of threads, for three months remained bedridden and only through the most assiduous medical care and nursing is she once again on the road to recovery.

The other cases, while not of so extreme a degree of severity, were nevertheless, for a while at least, of alarming nature involving cardiac complications and a general inhibition of nervous function, requiring weeks of watchful supervision before their respective conditions, physical and mental, became as satisfactory as they had been prior to the extractions. The number of teeth extracted at one sitting varied from two to six.

In studying these cases from clinical and pathological standpoints several important conclusions were reached. In each case it appeared that the patient was suffering from a combination of surgical shock and bacterial absorption, the former notwithstanding that the operations were performed under successfully administered conduction anesthesia. It was further brought out that these patients were to some extent exhausted or somewhat below par from disease, mental preoccupation or physical overwork and that the shock was not caused by surgical trauma, but by impressions received prior and subsequently to, but not during the surgical operations. The other feature of the post-surgical complication—the absorption of bacteria and their toxins—was the result of the relatively extensive surgical tissue traumatization incident to the extractions and curettements. In the presence of a high degree of vital defensiveness, the corollary of non-interference with nutritional functions, the extraction of several teeth at one sitting is not likely to result seriously to the patient, but the reverse of this hypothetical situation being the case the absorption upon a large scale of bacteria and their toxins results in most cases in those symptoms peculiar to the organisms which produce them, but the most serious manifestations are upon the nervous system and upon the heart.

The writer has not in mind the wholesale condemnation of multiple extractions—anything over one tooth—

for frequently such operations are performed without the subsequent development of noticeable complications. The aim of this narrative is to point out how aggravated and even fatal may be the after-results of the removal upon an extensive scale of infected teeth and adjacent areas in individuals whose resistance is below normal by reason of the existence of chronic maladies and nervous complications. There is sufficient clinical evidence to show conclusively that the extraction of even one single tooth is not under the abnormal conditions above outlined, a simple operation to be undertaken without any investigation at all of existing conditions. By some few operators and diagnosticians the extraction of diseased teeth is being approached from a different angle than appears to be the case with the vast majority of those who undertake these kind of operations. The extraction of one or more teeth embraces a consideration of a number of factors in addition to those connected with the actual operation incident to their removal. The need for more intense interest in the realms of pathological and clinical investigations in dentistry is very apparent in every department of professional endeavor.—Editorial *Pacific Gazette.*

---

Endowment for Hopkins University School of Hygiene. The magnificent gift of $6,000,000 by the Rockefeller Foundation to the Johns Hopkins University for endowment of its School of Hygiene represents a recognition by the foundation of the great strides that preventive medicine has made in the last decade. When the Hopkins school was opened in 1918, the Rockefeller Foundation consented to make annual contributions for a series of years, but the school had not an assured and definite income on which it could build for the future. Of the present gift, $1,000,000 is to be utilized for the construction of a building, plans for which have already been drawn, and the remaining $5,000,000 is to constitute a permanent endowment which is expected to yield an annual income of $250,000 for maintenance. Since its establishment, the School of Hygiene has exercised a great influence in advancing the cause of preventive medicine. At present, there are enrolled 131 students, who include representatives from twenty-seven states and ten foreign countries. Dr. William H. Welch, director, and Dr. William H. Howell, assistant director, have laid emphasis on the training of men and women competent to accept positions as health officials in communities of importance. In addition to outlining a regular course of study leading to the degrees of Doctor of Public Health, Doctor of Science in Hygiene, and Bachelor of Science in Hygiene, it is planned to give short courses or institutes for health workers already in service. It is interesting to learn also that the state of Maryland has encouraged the giving of such contributions as the Rockefeller Foundation has made to the Hopkins school by passing a law exempting such gifts from taxation.—*Journal A. M. A.*

---

The Oral Prophylaxis Chart. A real tangible step in reaching the goal of preventive medicine is made by the adoption and use of the "Oral Prophylaxis Chart." It means that prophylactic dentistry is here and is here to stay. This work is pleasing, inspiring and gratifying. The chart serves as a guide in establishing and maintaining a condition in the human mouth wherein disease will not prevail. The mouths of patients receiving this attention are distinctive in their beauty. This is the definite goal toward which we must strive with our patients.

They are seen at one, two or three month intervals, and areas not under perfect control by the patient are drawn to their attention in order that they may concentrate on them, thereby further raising the general tone of the mouth until an almost inconceivable condition of oral health is reached. This, of course, implies that proper restorative operations must be performed.—Extract from a paper by Drs. Best and Waldron, in *Journal N. D. A.*

### PATHOLOGICAL CONDITIONS OF PULPS AND PULPLESS TEETH

*Classification and Principles of Practice*—Best and Waldron *

| Conditions Present | Disposition in Adult Cases | |
|---|---|---|
| | Well Patients | Sick Patients |
| (Class I) Pulpless molars and bicuspids with definite periapical atrophy | Extract | Extract |
| (Class II) Pulpless molars and biscuspids without definite periapical atrophy | Retain and keep under observation, X-ray once a year | Such teeth should be extracted only after consultation with physician in charge |
| (Class III) Pulpless centrals, laterals and cuspids with definite periapical atrophy | Extract or, if area of atrophy is not too large, resect root end and curette. Keep under observation, X-ray twice a year | Extract |
| (Class IV) Pulpless centrals, laterals and cuspids without definite periapical atrophy | Retain and keep under observation, X-ray once a year | Extract only after consultation with physician in charge |
| (Class V) Molars and bicuspids containing non-vital pulps showing periapical atrophy | Extract | Extract |
| (Class VI) Molars and bicuspids containing non-vital pulps not showing periapical atrophy | Retain, open, cleanse, sterilize and seal canals. Keep under observation, X-ray once a year | Extract |
| (Class VII) Centrals, laterals and cuspids, containing non-vital pulps, with definite pathology | If area of pathology is not too large, open, cleanse, seal canal. Resect, X-ray twice a year | Extract |

| | | |
|---|---|---|
| (Class VIII) Centrals, laterals and cuspids, containing non-vital pulps, without definite pathology | Save and keep under observation, X-ray once a year | Such teeth should be extracted only after consultation with physician in charge. |
| (Class IX) Removal of vital pulps | Permissible in three classes * | Permissible in Classes I* and II* but not desirable |
| | Disposition in Children's Cases | |
| (Class X) Deciduous teeth containing gangrenous pulps | Extract | Extract |
| (Class XI) Deciduous teeth with exposed pulps | Make every effort to save pulp; if impossible, extract and retain space | Make every effort to save pulp; if impossible, extract and retain space |
| (Class XII) Permanent teeth with incompletely developed roots containing gangrenous pulps | Extract | Extract |
| (Class XIII) Permanent teeth with incompletely developed roots and pulps exposed | Make every effort to save pulp; if possible, extract | Make every effort to save pulp; if possible, extract |
| (Class XIV) Permanent teeth with roots completely formed and pulps exposed | See Class IX | See Class IX |

* Three classes for the removal of vital pulps: (1) permanent teeth where, as the result of accident or caries, the pulp has been either injured or exposed and can not be saved; (2) permanent teeth where pathological changes in the pulp are causing such intense pain that the result will be the loss of the tooth or pulp; (3) vital permanent teeth in which an insufficient amount of the crown of the tooth is left for anchorage purposes.

No tooth on which root-canal work has been done recently should be used as abutments for permanent bridge until they have been kept under observation for at least six months.

There is insufficient evidence at the present time to justify us in removing non-vital teeth, even in sick patients where the pericemental membrane and pericemental lamella are normal radiographically.—Best and Waldron, in *Journal N. D. A.*

Our Knowledge of Vitamins. Commenting on the trend of medical research concerning vitamins, the latest report of the British Medical Research Council says:

The present situation is a curious one, upon which posterity will probably look back with great interest. We still have almost no knowledge of the nature of these elusive food substances or of their mode of action, but we have gained empirical knowledge already of the greatest practical value for the prevention of scurvy and of other grave diseases and for the promotion of health and beauty in the population.

This statement, it will be noted, emphasizes the foundation on which rests our present use of vitamins. From time to time *The Journal* has commented on our lack of actual knowledge of these mysterious substances, emphasizing particularly the generally accepted fact that the taking of a well-balanced diet results in providing the individual with such vitamins as are necessary to his growth and nutrition. Last week appeared a brief report of a meeting of the Chicago Medical Society devoted to this subject, and it was gratifying to have the conservative view which *The Journal* has emphasized substantiated by many of those who took part in the discussion. Moreover, the *British Medical Journal*, in its leading editorial for February 11th, reiterates that an abundant supply of vitamins exists in all fresh vegetables, and that a considerable quantity occurs in milk and meat, provided the latter substances are obtained from animals fed on fresh foods. ''A normal adult,'' it says, ''living on an ordinary diet containing a reasonable proportion of fresh vegetables is, therefore, certain of obtaining a plentiful supply of vitamins.'' Of all the mass of evidence which has accumulated relative to these substances, this fact is the point of greatest importance. It is, however, very unfortunately, the one point which those commercially inclined are unwilling to recognize.—*Journal A. M. A.*

